# P-1104. Multidrug-Resistant Organism (MDRO)-Contaminated Mobile Equipment Networks in Skilled Nursing Facilities

**DOI:** 10.1093/ofid/ofaf695.1299

**Published:** 2026-01-11

**Authors:** Lindsay D Visnovsky, Molly Leecaster, George G Vega Yon, Catherine M Loc-Carrillo, Egenia Dorsan, Tavis Huber, Styn M Jamu, Kristina Stratford, Matthew H Samore, Frank A Drews

**Affiliations:** University of Utah School of Medicine, Salt Lake City, UT; University of Utah, Salt Lake City, Utah; Spencer Fox Eccles School of Medicine, University of Utah & IDEAS Center of Innovation, VA Salt Lake City Health Care System, Salt Lake City, Utah; Spencer Fox Eccles School of Medicine, University of Utah & IDEAS Center of Innovation, VA Salt Lake City Health Care System, Salt Lake City, Utah; Spencer Fox Eccles School of Medicine, University of Utah & IDEAS Center of Innovation, VA Salt Lake City Health Care System, Salt Lake City, Utah; Spencer Fox Eccles School of Medicine, University of Utah & IDEAS Center of Innovation, VA Salt Lake City Health Care System, Salt Lake City, Utah; U.S. Department of veterans Affairs. VA SLC, Utah, Salt Lake City, Utah; University of Utah, Salt Lake City, Utah; University of Utah, Salt Lake City, Utah; Spencer Fox Eccles School of Medicine, University of Utah & IDEAS Center of Innovation, VA Salt Lake City Health Care System, Salt Lake City, Utah

## Abstract

**Background:**

Multidrug-resistant organisms (MDROs) are prevalent in skilled nursing facilities (SNFs). Mobile equipment has been linked to outbreaks but its role in MDRO endemicity is less understood.

**Figure 1.**
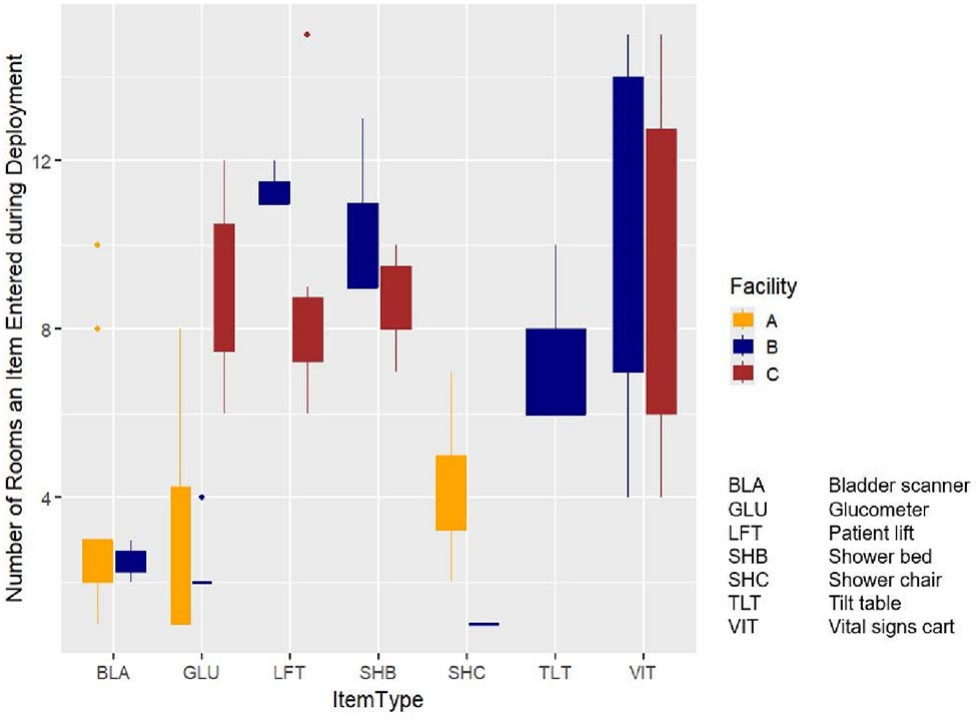
Median Number of Patient Rooms Visited (Degree) of Each Shared Equipment Item, by Facility* *Note: Due to size limitations, a wireless sensor was not attached to the CO2 monitors, although microbiological sampling was conducted. Among the 8 potential types of equipment sampled, each facility had a unique set of equipment that was mobile and shared between patients. See Figure 2 for a facility-specific list of shared mobile equipment. Boxplots are displayed only if the item type was mobile (not a patient room composite surface) and was shared in the facility.

**Figure 2.**
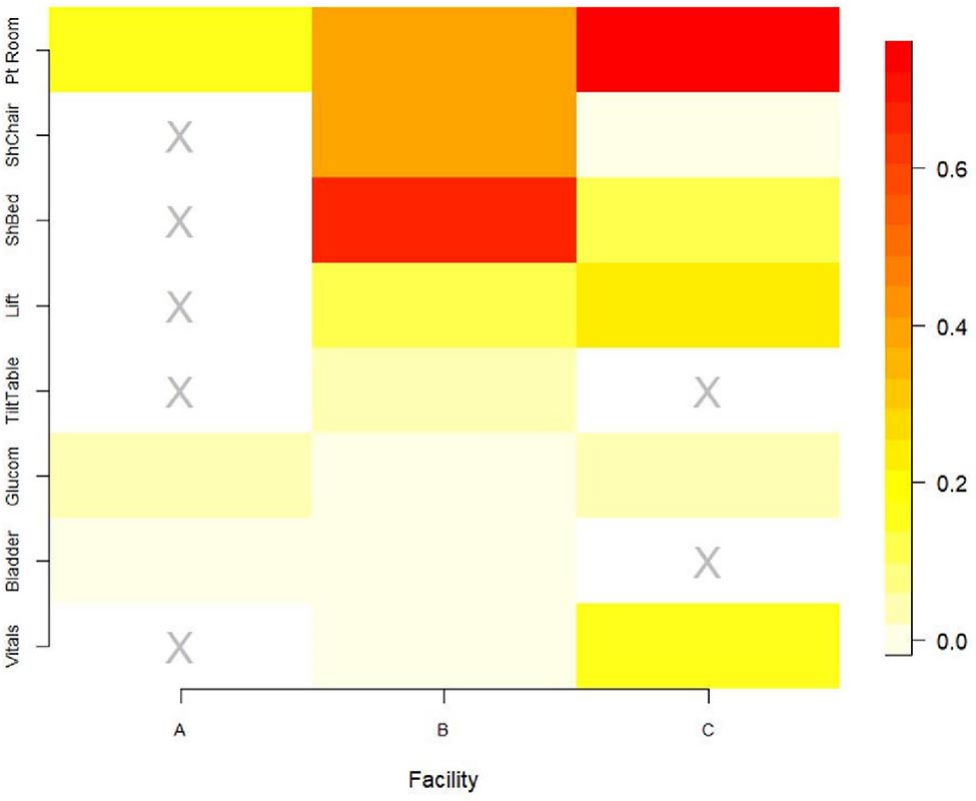
Proportion of patient room composite* samples (N=174 room samples, all sites) and shared equipment† items (N=473 equipment samples, all sites) positive for any multidrug-resistant organism (MDRO).‡ *Composite samples taken in patient rooms included the following surfaces: Facility A—bedrail, computer keyboard, IV pole, lift button, and vital signs touchscreen; Facility B—armchair, bedrail, pulse oximeter, table, ventilator; Facility C—armchair, bed remote, pulse oximeter, table, ventilator. Note: the CO2 monitor (mobile equipment item) was sampled 7 times and no MDROs were detected. †Some facilities had dedicated equipment for each room, precluding sampling as a mobile, shared surface. The gray “X” above indicates that the equipment item was not shared between rooms at that facility. ‡”Any MDRO” here refers to at least one of the multidrug-resistant organisms (MDROs) we tested for, including: methicillin-resistant Staphylococcus aureus, vancomycin-resistant Enterococcus, extended-spectrum β-lactamase (ESBL)-producing Enterobacteriaceae, or multidrug-resistant Acinetobacter species.

**Methods:**

We conducted 2 waves of 4-day microbiological sampling 3 to 6 months apart in 3 ventilator-capable SNFs (1 academic affiliate, 2 community-based). We collected patient room composite samples at shift start; up to 8 types of shared equipment were sampled at start and end of sampling day and after observed use. Samples were enriched and plated on selective media (for methicillin-resistant *Staphylococcus aureus*, vancomycin-resistant Enterococcus, extended-spectrum β-lactamase producing Enterobacteriaceae, and multidrug-resistant Acinetobacter species). Presumptive MDRO positives were MALDI-confirmed. Wireless sensors in patient rooms and attached to shared equipment recorded equipment location and movement, allowing creation of bipartite networks (nodes: rooms and equipment). Two rooms were connected when the same equipment item visited both rooms. We used Exponential Family Random Graph Models to analyze network structure and test if: 1) a room was more likely to have an MDRO detected if connected by equipment to another room with the same MDRO and 2) MDRO detection frequency differed by facility.

**Table 1.**
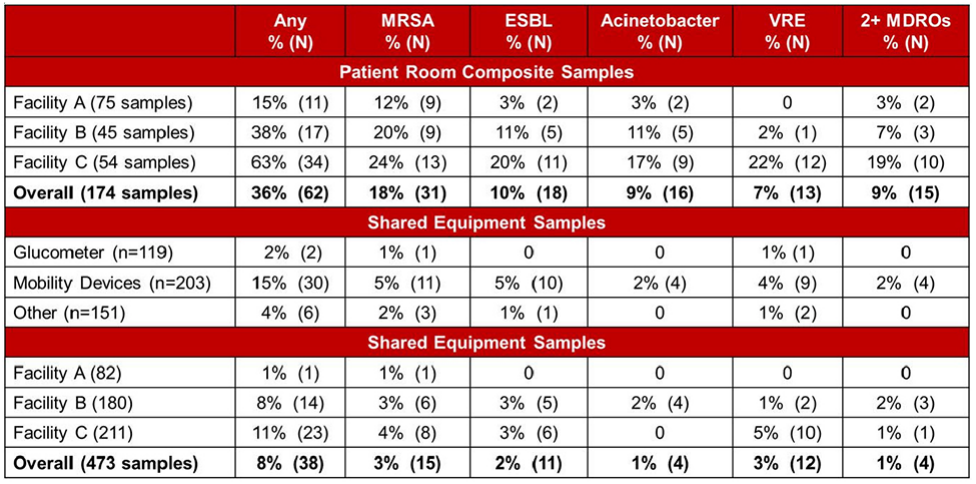
Facility-specific percentage (N) of MDRO-positive patient room samples (N=174), stratified by organism (top panel) and percentage (N) of MDRO-positive samples by shared equipment type (middle panel) and facility (bottom), N=473. Mobility devices include: shower bed, shower chair, Hoyer lift, and tilt table. Other devices include: bladder scanner, vital signs cart, and CO2 monitor.

**Results:**

8 types of mobile equipment were studied but only glucometers were shared items at all facilities (Figure 1). Equipment degree (the number of different rooms an item visited during deployment) varied substantially (Figure 1). MDRO contamination depended on facility and equipment type (Figure 2). ESBLs and MRSA were the most common MDROs on room surfaces and mobile equipment. Contamination was low in facility A relative to other facilities (Table 1). Vital signs carts had a high median degree (12) but were rarely contaminated (6%, n=6). Shower beds also had high median degree (9) but were often contaminated (25%, n=13) in the two facilities that used them. Network analysis of facility B found that rooms connected by shared equipment were more likely to be contaminated by the same MDRO than unconnected rooms (p< 0.05).

**Conclusion:**

Shared equipment in SNFs moves between patient rooms and is often MDRO-contaminated. Future work should study the role of shared equipment in MDRO transmission.

**Disclosures:**

All Authors: No reported disclosures

